# Alpha-ketoglutarate extends *Drosophila* lifespan by inhibiting mTOR and activating AMPK

**DOI:** 10.18632/aging.102045

**Published:** 2019-06-26

**Authors:** Yuan Su, Tao Wang, Nan Wu, Diyan Li, Xiaolan Fan, Zhongxian Xu, Shailendra Kumar Mishra, Mingyao Yang

**Affiliations:** 1 Animal Genetic Resources Exploration and Innovation Key Laboratory of Sichuan Province, Sichuan Agricultural University, Chengdu, P.R. China

**Keywords:** AKG, *Drosophila*, lifespan, AMPK, mTOR

## Abstract

Alpha-ketoglutarate (AKG) is a key metabolite of the tricarboxylic acid (TCA) cycle, an essential process influencing the mitochondrial oxidative respiration rate. Recent studies have shown that dietary AKG reduces mTOR pathway activation by inhibiting ATP synthase, thereby extending the lifespan of nematodes. Although AKG also extends lifespan in fruit flies, the antiaging mechanisms of AKG in these organisms remain unclear. In the present study, we explored changes in gene expression associated with the extension of *Drosophila* lifespan mediated by dietary AKG. Supplementation of the flies’ diets with 5 μM AKG extended their lifespan but reduced their reproductive performance. Dietary AKG also enhanced vertical climbing ability, but did not protect against oxidative stress or increase tolerance to starvation. AKG-reared flies were resistant to heat stress and demonstrated higher expression of heat shock protein genes (*Hsp22* and *Hsp70*) than control flies. In addition, AKG significantly upregulated mRNA expression of *cry*, *FoxO*, *HNF4*, *p300*, *Sirt1* and *AMPKα*, and downregulated expression of *HDAC4*, *PI3K*, *TORC*, *PGC*, and *SREBP*. The metabolic effects of AKG supplementation included a reduction in the ATP/ADP ratio and increased autophagy. Collectively, these observations indicate that AKG extends *Drosophila* lifespan by activating AMPK signaling and inhibiting the mTOR pathway.

## INTRODUCTION

In recent years, many studies have focused on diet composition and its effects on aging phenotypes [1]. Along with several natural products and synthetic drugs, there is now strong evidence that nutrition can be used to treat age-related diseases and delay aging in humans [2]. Alpha-ketoglutarate (AKG) is a crucial intermediate metabolite in the tricarboxylic acid (TCA) cycle and plays a key role in cellular energy metabolism [3]. AKG is water soluble, nontoxic, and highly stable in aqueous solutions. It is one of the nutritional ingredients in sports drinks, and a component of liver function test kits. In adulthood, administration of AKG has been found to promote longevity, although it was ineffective in aged organisms [4]. During cellular metabolism, AKG produced by the TCA cycle can be used to synthesize several amino acids. Therefore, during AKG deficiency, dietary supplementation with AKG can compensate this deficit. In experiments carried out on pigs, Kristensen et al. [5] showed that dietary supplementation with 1.25% AKG led to the synthesis of proline, a main component of collagen, in intestinal absorptive cells. Dąbek et al. [6] found that AKG is better absorbed in the upper part of the small intestine than in the distal section.

In the past decade, more than 400 different drugs and compounds in the DrugAge database (http://genomics.senescence.info/drugs/) have been reported to extend lifespan and reduce age-related diseases in model organisms. Such compounds include rapamycin [7, 8] metformin [9] and the anticancer agent trametinib [10]. The effects of these drugs are commonly associated with down-regulation of the insulin/insulin-like growth factor-1 signaling (IIS) pathway and the mechanistic target of rapamycin (mTOR) pathway [11, 12]. A recent study reported that by inhibiting ATP synthase and TOR, AKG extended the lifespan of adult *C. elegans* [4]. This was paralleled by reduced ATP levels and oxygen intake, and increased autophagy in both *C. elegans* and mammalian cells. Interestingly, this study demonstrated also that AKG delayed age-related phenotypic changes, such as declines in rapid coordinated body movement [4]. Furthermore, earlier studies had suggested that AKG extends longevity primarily by inhibiting ATP synthase in yeast [13], worms [14, 15], flies [16, 17], and mice [18] and that this effect partially depends on AMPK and FoxO.

A recent study evaluated the effects of an AKG-supplemented diet on lifespan and functional aging in the Canton-S strain of *Drosophila melanogaster* [19]. The study revealed both dose- and sex-dependent effects of dietary AKG. In males, dietary supplementation with 10 mM AKG extended maximum lifespan, whereas in females 10 and 20 mM AKG increased both median and maximum lifespan [19]. Other studies also showed that dietary AKG can increase amino acid and protein levels, lipid absorption, and antioxidant defenses in fruit flies [20]. Recently, the effect of dietary AKG was investigated to elucidate the mechanism of aluminum toxicity in *D. melanogaster,* and it was found that AKG could alleviate multiple metabolic effects of high aluminum exposure [21]. These findings suggested that AKG can be utilized as an antidote for aluminum-containing compounds that are harmful to human health due to their widespread household and industrial use. Although the effect of AKG on *Drosophila* lifespan has been elucidated in recent years, its molecular basis remains unclear. Therefore, we used *Drosophila* as a research subject to further explore the antiaging effect of AKG and to shed light on its genetic mechanisms. We believe our findings will be significant to the fields of aging and age-related disease research.

## RESULTS

### AKG extends *Drosophila* lifespan

**Figure 1:**
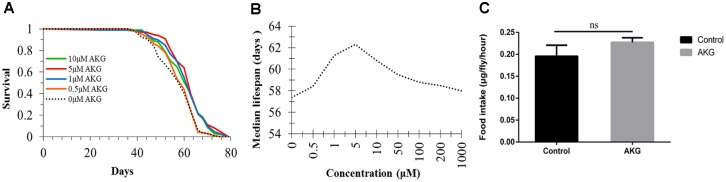
**Effect of AKG on *Drosophila* lifespan.** (**A**) Effects of low AKG concentrations on *Drosophila* lifespan. Treatment with low-dose AKG (0.5, 1, 5, and 10 μM) extended the lifespan of W^Dah^ females compared to control flies on 1x sugar/yeast/agar (SYA) food (1 μM vs 0 μM AKG, *p* < 0.001; 5 μM vs 0 μM AKG, *p* < 0.001; 10 μM vs 0 μM AKG, *p* = 0.002; log-rank test). (**B**) Effects of different concentrations of AKG on *Drosophila* median lifespan. (**C**) Food consumption measurement. There was no difference in food consumption between control and AKG-treated groups (ns = nonsignificant, *p* > 0.05, two-tailed Student’s *t*-tests).

First, we investigated the potential of dietary supplementation with AKG (from 0 to 1000 μM) to increase the lifespan of *Drosophila* (Supplementary Figure 1). Positive effects on lifespan were seen at intermediate doses of 1, 5, and 10 μM (Figure 1A); however, there were no effects below or above these concentrations, reflecting a typical nonmonotonic dose–response curve. The dose–response analysis showed that supplementation of flies’ diets with 5 μM AKG increased median lifespan by up to 8.54% (Figure 1A and 1B). In subsequent experiments, we selected 5 μM as the optimal dose. To assess whether the longevity benefits of AKG were indirectly caused by drug aversion and/or rejection that led to self-imposed dietary restriction (DR), we evaluated AKG’s effect on food intake. During a 1-hour feeding assay, AKG had no effect on food consumption (Figure 1C).

### Effects of AKG on fecundity, physical activity, and stress tolerance

**Figure 2:**
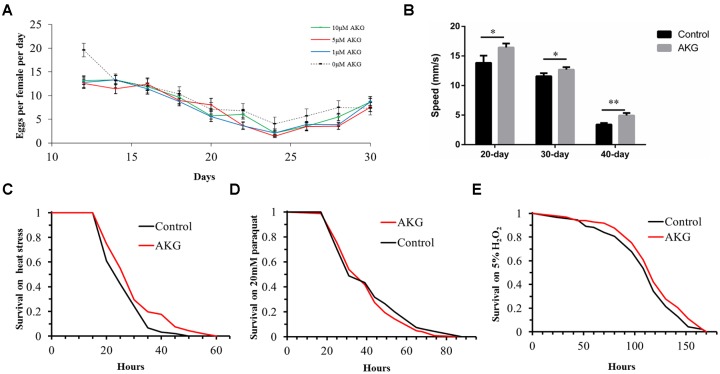
**Effects of AKG on reproduction, vertical climbing ability, and stress tolerance in w^Dah^ flies.** (**A**) Fecundity assay. AKG decreased fecundity at concentrations of 1 and 5 μM (1 μM vs 0 μM AKG, *p* = 0.0013; 5 μM vs 0 μM AKG, *p* = 0.005; Wilcoxon rank test) and had no significant effect at 10 μM (10 μM vs 0 μM AKG, *p* > 0.05, Wilcoxon rank test). (**B**) RING assay. AKG-supplementation increased vertical climbing ability at 3 time points (20, 30, and 40 days) compared with the control diet (*p < 0.05 and **p < 0.01, two-tailed Student’s *t*-test). (**C**) Heat stress assay. AKG-treated flies were significantly more resistant to 39°C heat stress than control flies (5 μM AKG vs control, *p* = 0.0023, log-rank test). (**D**) Paraquat stress assay. There were no differences in sensitivity to paraquat stress between control and AKG-treated flies (5 μM AKG vs control, *p* > 0.05, log-rank test). (**E**) H_2_O_2_ stress assay. There were no differences in sensitivity to H_2_O_2_ stress between control and AKG-treated flies (5 μM AKG vs control, *p* > 0.05, log-rank test). All data are shown as the mean ± standard error of the mean (SEM).

To evaluate potential mechanisms by which AKG prolonged lifespan, we assessed whether AKG affected fecundity and physical activity of flies during aging. We found that diets supplemented with 1 and 5 μM AKG significantly decreased fly fecundity, while 10 μM AKG had no significant effect (Figure 2A).

Then, the effect of AKG on flies’ body weight was assessed over 10 days, but no significant differences were observed between AKG-treated and control flies (Supplementary Figure 2). A rapid iterative negative geotaxis (RING) assay was performed to measure flies’ climbing ability (vertical locomotion). As expected, climbing ability declined with age in both groups. However, at days 20, 30, and 40, flies fed AKG climbed significantly higher than control flies (Figure 2B), indicating that AKG improved climbing ability and enhanced physical activity.

Furthermore, we examined whether AKG-induced longevity could be in part attributable to enhanced stress tolerance. For the first 10 days of adulthood, the flies were fed diets supplemented with AKG (or control diets) and tested for tolerance to heat stress, the superoxide generator paraquat, H_2_O_2_, desiccation, and starvation. We found that AKG significantly increased tolerance to heat stress (Figure 2C), and this effect was accompanied by significant increases in the expression of heat shock protein genes (*Hsp22* and *Hsp70*) compared with the control group (Supplementary Figure 3).

In contrast, AKG had no protective effect against paraquat (Figure 2D) or the oxidant H_2_O_2_ (Figure 2E). Accordingly, the expression of catalase (*CAT*) was not altered by AKG supplementation, whereas the expression of superoxide dismutase 1 (*SOD1*) was in fact inhibited (Supplementary Figure 4). Likewise, AKG did not affect tolerance to either desiccation or starvation (Supplementary Figure 5A, 5B). In addition, there were no significant differences in triacylglyceride (TAG) levels in flies treated with AKG compared with control flies (Supplementary Figure 5C), suggesting that lifespan extension was not associated with increased TAG levels.

### AKG supplementation activates the AMPK signaling pathway in *Drosophila*

**Figure 3:**
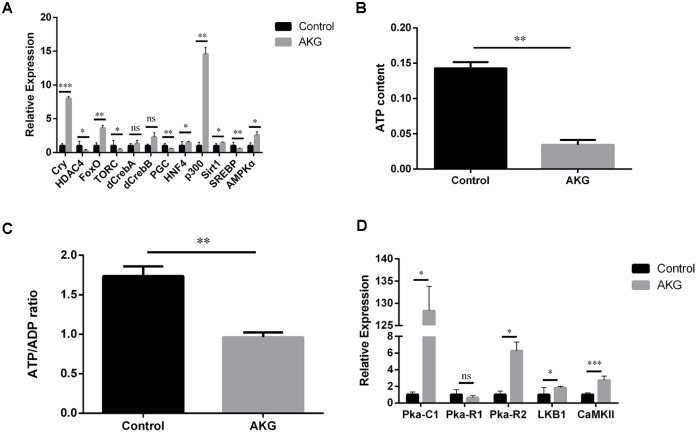
**AKG activates AMPK signaling and reduces ATP and the ATP/ADP ratio.** (**A**) Relative mRNA levels of genes downstream of AMPK (ns = nonsignificant, *p* > 0.05; *p < 0.05, **p < 0.01, and ***p < 0.001; two-tailed Student’s *t*-test). (**B**) AKG-supplementation decreases ATP content in fruit flies (**p < 0.01, two-tailed Student’s *t*-test). (**C**) AKG-supplementation decreases the ATP/ADP ratio (**p < 0.01, two-tailed Student’s *t*-test). (**D**) Relative mRNA levels of genes upstream of AMPK (ns = nonsignificant, *p* > 0.05; *p < 0.05, **p < 0.01, and ***p < 0.001; two-tailed Student’s *t*-test).

To examine whether the AKG-induced increase in *Drosophila* longevity was correlated with AMPK activation, we quantified relative steady-state levels of AMPK*α* and target mRNAs in flies fed AKG-supplemented or control diets. Transcript levels of *AMPKα* and AMPK downstream targets *cry*, *FoxO*, *HNF4*, *p300*, and *Sirt1* were significantly increased, while those of *HDAC4*, *TORC*, *PGC*, and *SREBP* were significantly decreased, in AKG-fed flies compared to controls (Figure 3A). However, the expression of *CREB* genes (*dCREB-A* and *dCREB-B*) was comparable between groups. These results suggest that AKG supplementation induces AMPK activation and AMPK-mediated changes in gene expression in *Drosophila*.

Next, to verify that the increased longevity of AKG-fed flies is related to changes in cellular energy status, we measured ATP content and the ATP/ADP ratio. Consistent with increased AMPK expression, a reduction in both total ATP content and in the ATP/ADP ratio was observed in flies supplemented with AKG (Figure 3B, 3C). We next assessed the expression of three PKA subfamily genes whose products are upstream regulators of AMPK activity. Of these, *Pka-C1* and *Pka-R2* were significantly increased, while the expression of *Pka-R1* was unaffected (Figure 3D). These findings indicate that AKG stimulated *PKA* expression, which might activate AMPK by enhancing the expression of *LKB1* (see below). Meanwhile, the expression of *CaMKII*, another AMPK-activating enzyme, was also significantly increased after AKG treatment (Figure 3D).

### AKG supplementation inhibits the mTOR signaling pathway

**Figure 4:**
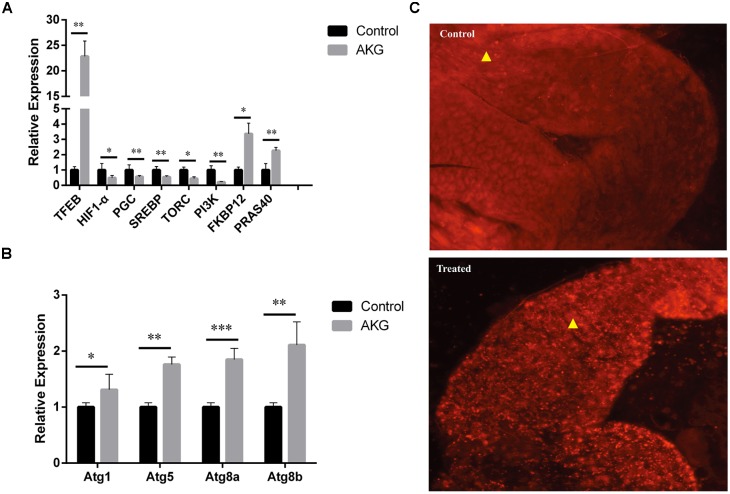
**AKG supplementation increases autophagy.** (**A**) Relative mRNA expression of genes downstream and upstream of mTOR (*p < 0.05 and **p < 0.01, two-tailed Student’s *t*-test). (**B**) Relative mRNA expression of autophagy-related genes (*p < 0.05, **p < 0.01, and ***p < 0.001, two-tailed Student’s *t*-test). (**C**) Phagosome staining. Representative fluorescence images of fly midguts stained with LysoTracker Red. Midguts of control flies (upper image) and AKG-treated flies (lower image) are shown.

The TOR signaling pathway is a critical mediator of cell metabolism and aging. Therefore, we investigated if changes in mTOR pathway outputs were consistent with the effects of AKG on *Drosophila* lifespan. We found that the expression of *PGC*, *SREBP*, and the upstream genes *PI3K* and *TORC* was significantly inhibited, while that of *FKBP12* and *PRAS40* significantly upregulated, by AKG treatment (Figure 4A).

It is well known that inhibition of mTOR can suppress protein synthesis and cell proliferation and increase autophagy. Consistent with the latter, we observed that AKG increased the expression of *TFEB*, which regulates the production of autophagosomes and lysosomes. Moreover, transcription levels of the autophagy-related genes *Atg*1, *Atg*5, *Atg*8a, and *Atg*8b were also significantly upregulated after AKG supplementation (Figure 4B). Meanwhile, phagosome staining in vitro confirmed increased phagosome formation in AKG-reared flies (Figure 4C).

### AKG modifies the expression of genes associated with the Warburg effect and the JNK signaling pathway

**Figure 5:**
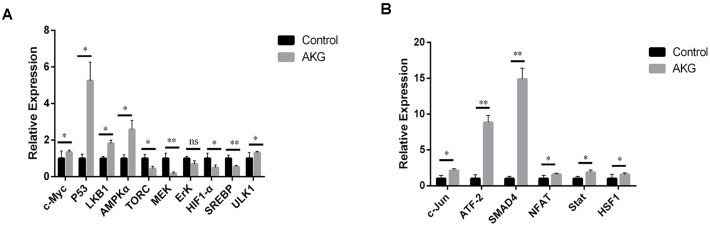
**AKG inhibits the Warburg effect and activates JNK signaling.** (**A**) Relative mRNA expression of downstream and upstream genes involved in the Warburg effect (ns = nonsignificant, *p* > 0.05; *p < 0.05 and **p < 0.01; two-tailed Student’s *t*-test). (**B**) Relative mRNA expression of downstream and upstream genes in the JNK signaling pathway (*p < 0.05 and **p < 0.01, two-tailed Student’s *t*-test).

AKG-reared flies exhibited significant upregulation of *c-myc*, *P53*, *LKB1*, AMPKα, and *ULK1* (Figure 5A). These findings further prove that AKG increases the expression of tumor suppressor genes, inhibits the Warburg effect, and enhances physiological resistance. On the other hand, the expression of protein clearance-related genes further indicates that AKG activates AMPK and inhibits mTOR. We also assessed the fatty acid oxidation pathway by analyzing *MEK* and *ErK* expression. There was no apparent increase in the activation of *MEK1/2* and *ErK1/2* following AKG supplementation, suggesting that these enzymes may not be the only ones involved in the *MEK* and *ErK* pathways; perhaps other factors increase *MEK1/2* and *ErK1/2* kinase activity, thus increasing *c-Myc* expression.

The C-jun N-terminal kinase (JNK) pathway, also known as the stress-activated protein kinase (SAPK) pathway, is an important branch of the MAPK pathway regulating cell cycle progression, differentiation, apoptosis, and cellular stress. We found that most genes downstream of *JNK*, including *NFAT (NFAT4* and *NFATC1)* and *Stat3*, were markedly upregulated by AKG supplementation (Figure 5B). These findings suggest that AKG supplementation activates the *JNK* pathway.

## DISCUSSION

Changes in the levels of AKG, an endogenous intermediary metabolite in the TCA cycle, can directly affect TCA fluxes and energy conversion processes and may influence numerous health conditions, such as autoimmune disorders and cancer [22, 23]. In recent years, studies have reported that AKG intake can inhibit mTOR and thus extend the lifespan of nematodes. Fruit flies, which were used in this study, are structurally different from nematodes and express many genes homologous to those associated with human diseases. Here, we investigated the effect of AKG on *Drosophila* lifespan and its potential mechanisms of action. We found that the lifespan-increasing effect of AKG was accompanied by, and consistent with, gene expression changes within the AMPK, mTOR, and JNK pathways.

Our results showed that AKG increased the average and maximum lifespan of flies by 8% and 15%, respectively, at a dose of 5 μM, without overt developmental toxicity. However, our findings are slightly different from those of previous studies that have used 10 mM AKG [19, 20]. Developmental investigations have shown that AKG greatly extends lifespan but reduces also fecundity, two parallel effects described as well for many other longevity-related drugs. Meanwhile, we found that AKG enhanced heat stress resistance and physical activity, while antioxidant and antistarvation responses were unchanged. These findings are consistent with those of previous studies in which dietary AKG was found to prolong lifespan and improve physical performance, although reducing fecundity in fruit flies.

AMPK activity is regulated by the relative levels of ATP and AMP, both of which bind competitively with its γ subunit [24]. Activated AMPK inhibits lipogenesis and glycogen and protein synthesis by targeting the ChREBP/SREBP1c, TIF-1A, mTORC1, and ACC1 signaling pathways [25–27]. Combined with those of previous studies, our findings indicate that the mechanism by which AKG extends lifespan involves reductions in both ATP levels and the ATP/ADP ratio, with subsequent activation of AMPK. Upon performing further RT-PCR analyses, several major transcription factors targeted by AMPK were found to be differentially regulated. Notably, there were also significant increases in *PKA* and *LKB1*, two major upstream kinases of AMPK. It has been suggested that *HNF4α* is a downstream target of *AMPKα* and that increased *HNF4α* levels, as detected in our AKG-fed flies, indicate AMPK activation [28]. Studies also showed that *PGC-1α*, found to be downregulated by AKG in our study, acts as a coactivator for several transcription factors involved in mitochondrial biogenesis and oxidative metabolism and is associated with an AMPK signaling network that enhances its deacetylation by *SIRT1* [29]. On the other hand, AKG can directly bind and inhibit the ATP synthase subunit β [4], which is consisting with the decrease in ATP levels observed in our study and substantial evidence previously obtained in several organisms [13–18].

The mTOR pathway is a highly conserved nutrient-sensing pathway that regulates cell metabolism as well as lifespan in response to various stresses, growth factors, and changes in cellular energy levels. Transcription factors in the mTOR pathway were inhibited in AKG-treated flies, suggesting that AKG decreases TOR pathway activity by inhibiting upstream transcription of the *HIF1-α*, *PGC*, *SREBP*, *TORC1*, and *PI3K* genes. However, the involvement of other signaling elements in this process is highly likely. The mRNA levels of autophagy-related genes and the number of phagosomes were also significantly increased in flies fed AKG, indicating increased autophagy. Our results are thus consistent with inhibition of TOR leading to activation of the autophagy pathway [12]. We also explored the expression of genes involved in some key signaling pathways that affect systemic metabolism, including those mediating the Warburg effect and members of the JNK pathway. Although *MEK1/2* and *ERK1/2* expression showed no apparent increase, *c-Myc* transcript levels were up-regulated by AKG, suggesting augmented glycolysis and fatty acid oxidation. In addition, the up-regulation of the tumor suppressor genes *P53* and *LKB1* by AKG treatment may indicate reduced susceptibility to cancer; although further studies are needed to validate this conjecture, it is now well established that abnormal metabolism of AKG can lead to the production of 2-hydroxyglutarate (2-HG) and promote oncogenic transformation [22].

In previous studies, we conducted transcriptome analyses of reproductive performance in chickens and other vertebrates [30, 31]. Through high-throughput sequencing, we detected a large number of differentially expressed genes, such as follicle-stimulating hormone (*FSHB*), gonadotropin-releasing hormone (*GnRH*), and prolactin (*PRL*), as well as several microRNAs (*gga-mir-34c-3p*, *gga-mir-122-5p*, *gga-mir-1683*, etc.) related to fecundity. As common model animals, flies express a large number of genes that are homologous to those found in vertebrates. We found that invertebrates and vertebrates have certain conserved mechanisms to control fecundity by extending lifespan and that these mechanisms can be regulated through the AMPK, mTOR, and JNK signaling pathways. Since flies have gene regulatory networks and signal transduction pathways similar to those found in vertebrates, experimental results from flies can provide references for studies on vertebrates.

## MATERIALS AND METHODS

### Standard laboratory food

The DR medium (1x SYA) contained 100 g/L yeast (1x; MP Biomedicals, USA), 50 g/L sucrose (Tate & Lyle, UK), 15 g/L agar (Sigma-Aldrich, UK), 30 mL/L Nipagin (Chemlink Specialities, UK) and 3 mL/L propionic acid (Sigma-Aldrich, UK). This diet and its method of preparation are described in Yang et al., 2016 [32]. The fully fed medium (2x SYA) was prepared in the same way except that it contained 200 g/L yeast.

### Experimental food

AKG (Sigma-Aldrich, UK) was dissolved in Milli-Q water to concentrations of 0.5, 1, 5, 10, 50, 100, 200, and 1000 μM and added to the SYA food. Milli-Q water without AKG was added to the control food.

### Fly lifespan

The wild-type Dahomey (w^Dah^) stock was collected in 1970 in Dahomey (now Benin). Experiments were conducted at 25°C under a 12-hour light/dark cycle at constant humidity of 65% [33, 34]. The flies were reared at a standard larval density of ~300 flies per bottle, and all enclosed adults were collected over a 12-hour period. After eclosion, the flies were allowed to mate for 48 hours, and then the females were separated under CO_2_ anesthesia for further experiments. The females were then randomly allocated to the experimental food treatment groups and housed in plastic vials containing food at a density of 10 flies per vial, with 10 vials per condition (n = 100). The flies were transferred to a fresh food source 3 times per week, during which any deaths and censors were recorded. All lifespan experiments were repeated at least twice. Fly survival was evaluated with the log-rank test.

### Food intake assay

One hundred female flies (10 flies per vial) per treatment (AKG or control) were reared in DR medium (1x SYA) containing 2.5% blue dye (wt/vol; Blue No. 1). After feeding for 1 hour, the flies were snap-frozen in liquid nitrogen and then homogenized in 200 mL of distilled water. An additional 800 mL of distilled water was added to the suspension, which was then filtered to remove debris and lipids through a 0.22-mm Millex filter (Millipore Corporation, Bedford, MA). The absorbance of each liquid sample was then measured at 629 nm (Thermo Scientific NanoDrop 2000c) [12, 35]. Statistical significance was determined using two-tailed Student’s *t*-tests.

### Measurement of fecundity

Eggs were collected every 2 days for 18- to 24-hour periods during the lifespan experiments. The number of eggs laid per vial at each time point was determined by eye using a light microscope. For each condition and each time point, 10 vials were counted. Each vial contained 10 flies. Fly fecundity was evaluated with the Wilcoxon rank test.

### Measurement of body weight and vertical climbing ability

Flies were reared in 10 vials (20 flies per vial; n = 200) containing an AKG-supplemented experimental diet or a control diet for 10 days. Flies were then collected under CO_2_ anesthesia and weighed. Vertical climbing ability was measured by RING assay, as described in Schriner et al. 2014 [36]. At the ages of 20, 30, and 40 days, 100 flies were placed in 10 vials for each treatment group. Each vial was gently shaken until all flies had been displaced to the bottom. The flies were permitted to climb for 4 s and then photographed. Flies in both groups (AKG-fed and control) were assayed simultaneously. The assay was repeated 5 times with independent groups of flies. The distance climbed was measured in Adobe Photoshop, and the speed was calculated based on the distance/4 s. Statistical analysis was performed with two-tailed Student’s *t*-tests.

### Stress assays

One hundred female flies (10 flies per vial) per treatment were fed for 10 days with an AKG-supplemented experimental diet or a control diet before being transferred to stress conditions. For the H_2_O_2_ and paraquat assays, flies were fed 5% H_2_O_2_ or 20 mM paraquat diluted in a 5% glucose solution supplied on filter paper, and deaths were recorded every 5 hours. For the heat stress assay, experimental flies were housed in empty vials at 39°C, and deaths were recorded every hour. For the desiccation assay, experimental flies were housed in empty vials, and deaths were recorded every 3 hours [28]. For the starvation assay, flies were housed in vials containing 1.5% agarose with no nutritional value to provide moisture, and deaths were recorded every day. Survival for all stress assays was determined by the log-rank test.

### Triacylglyceride (TAG) measurement

Flies were fed an AKG-supplemented experimental diet or a control diet for 10 days before collection, and each group was divided into six replicates of 10 flies. The flies were snap-frozen in liquid nitrogen and used for TAG content measurements according to the methods of a triglyceride assay kit (Nanjing Jiancheng Bioengineering Institute). Statistical analysis was performed with two-tailed Student’s *t*-tests.

### Measurement of ATP content and ATP/ADP ratio

Flies were fed an AKG-supplemented experimental diet or a control diet for 10 days. Each group was divided into six replicates of 10 flies. ATP content and ATP/ADP ratio were measured with an ATP content assay kit and an ATP/ADP ratio assay kit, respectively. Statistical analysis was performed with two-tailed Student’s *t*-tests.

### LysoTracker staining, imaging, and image analysis

Flies (n = 15) were reared on an AKG-supplemented diet or a control diet for 10 days. The methods for LysoTracker staining and dissection of complete guts have been described in our previous work [12]. All images were captured with a fluorescence microscope, and we used ImageJ to analyze the number of LysoTracker-stained puncta.

### Analysis of gene expression

Flies were fed for 10 days with an AKG-supplemented diet or a control diet and then frozen in liquid nitrogen for RNA quantification. Total RNA was extracted from 10 flies using TRIzol reagent, and reverse transcription was performed with a SuperScript II cDNA Synthesis Kit (Invitrogen). In addition, real-time quantitative PCR was performed using SYBR Green with RT-PCR primer pairs (Supplementary Table 1) on a Bio-Rad IQ5 (Thermo Fisher Scientific) instrument. Fold changes in expression were calculated using the 2^−ΔΔCt^ method and normalized to *actin* (housekeeping gene) levels. All measurements were performed in triplicate on each of three independent biological replicates, and data are presented as the mean ± standard deviation (SD). Statistical analysis was performed with two-tailed Student’s *t*-tests.

### Statistical analysis

Statistical analysis was performed using GraphPad Prism software version 6.0 (GraphPad Software Inc., La Jolla, CA, USA) and SPSS software version 17.0 (SPSS Inc., Chicago, IL, USA). The tests used and the sample sizes for each experiment are indicated in the corresponding figure legends and in Materials and Methods.

## CONCLUSION

In conclusion, our results demonstrated that AKG significantly extended the lifespan of flies but reduced their reproductive performance. Moreover, dietary supplementation with AKG significantly enhanced climbing ability and heat stress resistance but exerted no protective effects against oxidative stress. At the cellular level, AKG affected energy status by reducing the ATP/ADP ratio, and increased autophagy by promoting AMPK activity, repressing TOR signaling, and activating the JNK pathway.

## Supplementary Material

Supplementary Figures

Supplementary Table 1
