# Supplementary material for: Alpha-ketoglutarate extends Drosophila lifespan by inhibiting mTOR and activating AMPK
Source: Aging (Albany NY). 2019 Jun 26;11(12):4183–97. doi: 10.18632/aging.102045 (PMC6629006; doi:10.18632/aging.102045)
Supplement: Supplementary Figures [file aging-11-102045-s002.pdf]

SUPPLEMENTARY FIGURES

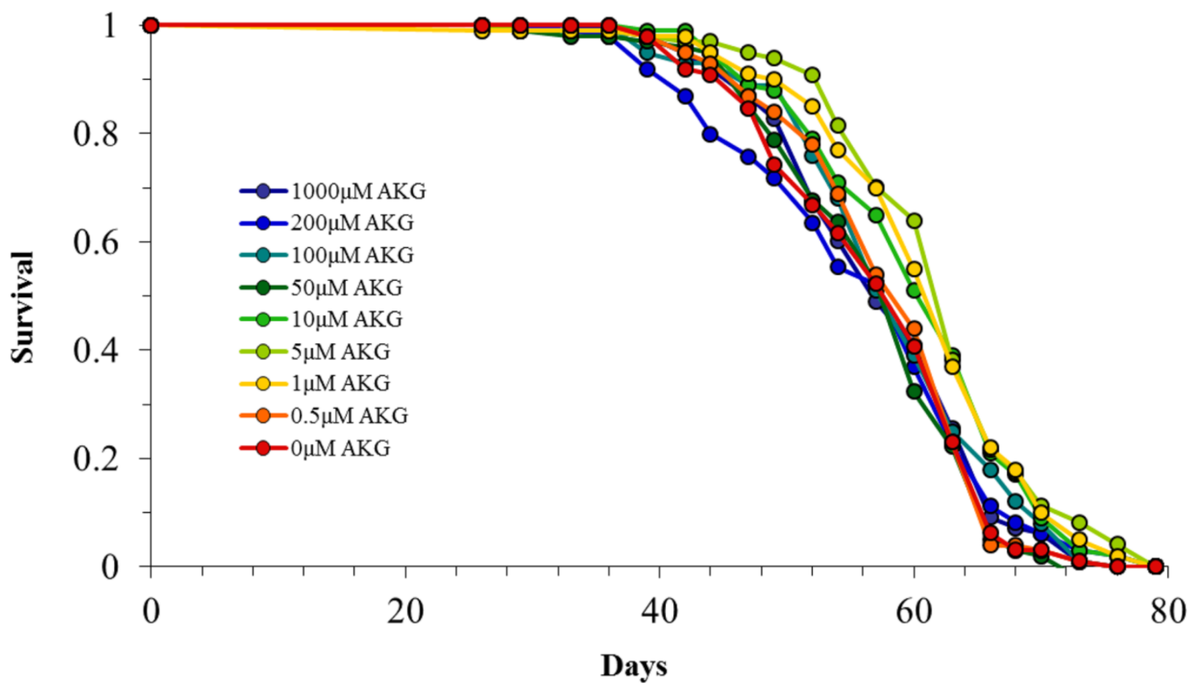

Supplementary Figure 1. Effects of different AKG concentrations (0, 0.5, 1, 5, 10, 50, 100, 200, and 1000 µM) on *Drosophila* lifespan.

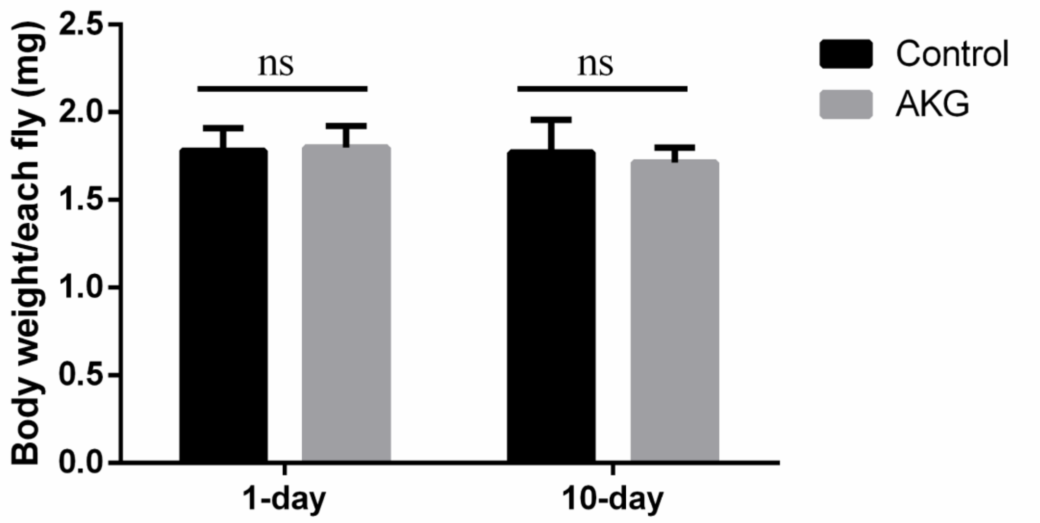

Supplementary Figure 2. AKG had no effect on the body weight of flies (10 days old) (ns = nonsignificant, *p* > 0.05, two-tailed Student's *t*-tests).

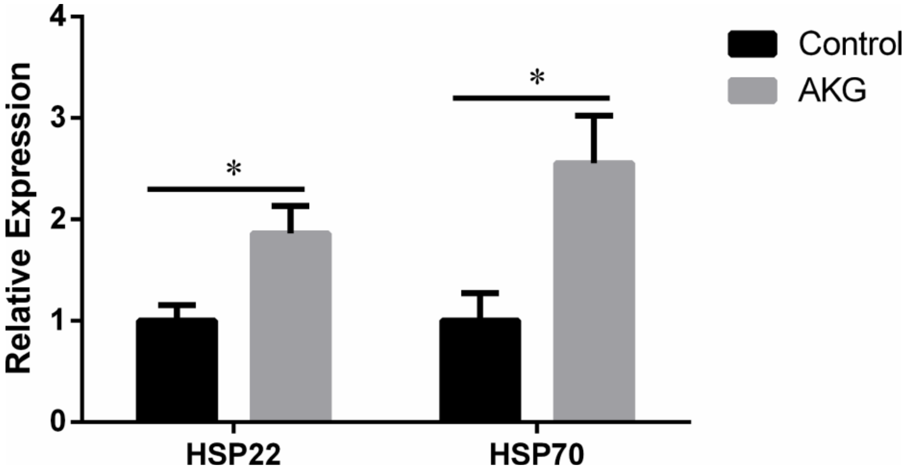

Supplementary Figure 3. Relative mRNA expression of *HSP22* and *HSP70* (*p < 0.05, two-tailed Student's *t*-tests).

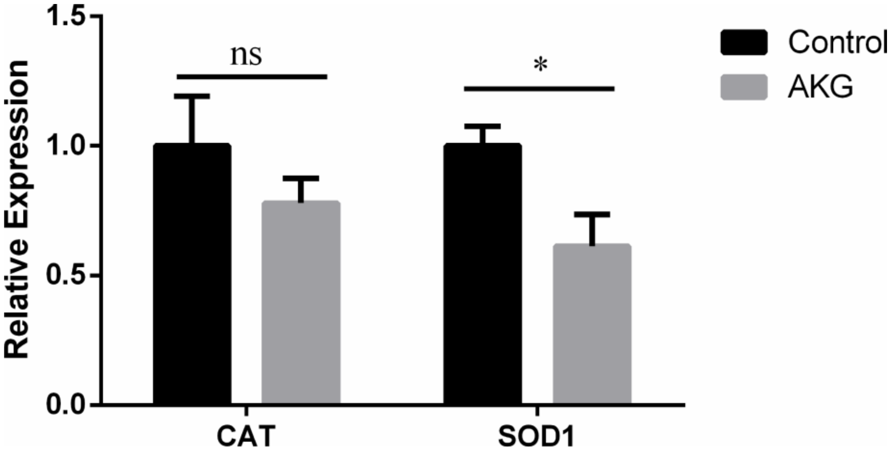

Supplementary Figure 4. Relative mRNA expression of *CAT* and *SOD1* (ns = nonsignificant, p > 0.05; *p < 0.05; two-tailed Student's t-*tests*).

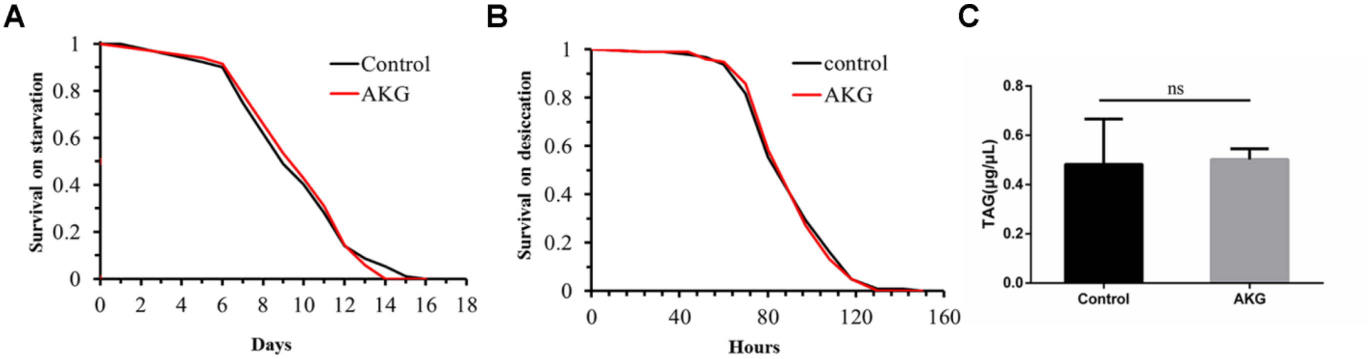

Supplementary Figure 5. Anti–oxidative stress effects of AKG on Drosophila. (**A**) There were no differences in sensitivity to starvation stress between control and AKG-treated flies (5 μM AKG vs control, $p > 0.05$, log-rank test). (**B**) There were no differences in sensitivity to desiccation stress between control and AKG-treated flies (5 μM AKG vs control, $p > 0.05$, log-rank test). (**C**) There were no differences in triacylglyceride (TAG) levels between control and AKG-treated flies (ns = nonsignificant, $p > 0.05$, two-tailed Student's t-tests).
