# Supplementary material for: Alpha-ketoglutarate extends Drosophila lifespan by inhibiting mTOR and activating AMPK
Source: Aging (Albany NY). 2019 Jun 26;11(12):4183–97. doi: 10.18632/aging.102045 (PMC6629006; doi:10.18632/aging.102045)
Supplement: Supplementary Table 1 [file aging-11-102045-s001.pdf]

SUPPLEMENTARY TABLE

Supplementary Table 1. Primers used for quantitative real-time PCR of aging-associated genes.

Gene	Primer (Forward)	Primer (Reverse)
Actin5C	CGGTATCGTTCTGGACTCCG	GCGGTGGTGGTGAAAGAGTA
HSP70	ACCAAGGGGTGTGCCCCAGA	CTTGGCCTTGCCCGTGCTCA
HSP22	TTGGCGGATGGCCGAGGAGA	AGCGCCACACTCCAAACGGG
CAT	ATGCGGCTTCCAATCAGTTGAT	CGAAGTGCGACATCTCATCCA
SOD1	ATGGTGGTTAAAGCTGTCTG	TCTTGGAGTCGGTGATGTT
Cry	CCACCGCTGACCTACCAAAT	GGAAGCCCATGTTGTCTCCA
HDAC4	CGAATGGCACAACGACAACG	GTTCGGTTCGGATGCTGTCT
Foxo	GCGCCTCTACTTTGATAGGAC	TATTGATGTCCAGCAGGCCG
TORC	CCAACGGTTACAACAAGCCG	TGGATCCCACCACACCATTG
CrebA	GACGTCAAAGATCCCACCGT	GATCTTGGCCTGGAGGCTTT
CrebB	GATCCCACGTACTACCTGTCC	TTTGGTTCTCTAGCACCGCC
PGC	CCTCGATGGCATCCTACGAC	TTGCTGGAAAACCTGGCAGA
Hnf4	CGACAATCCTCTGCCGCTAT	TCCAGGTCCAGACTATGCGA
P300	TGCGGCAGGAAGTACACATT	TCTATTTGAGGCGACACCGT
Sirt1	AACCAGGTGCCAGACACTAC	GTTGAGGCCAGAATTTCCGC
SREBP	TGGCTTCTACCAAGTGCCAG	CAAGAGCTGTTGCGTTGGAC
AMPKα	GGCCATCGCTTACCATCTGA	ATCGATGATTGGGGAACGGG
Pka-C1	CGGACTTCGGTTTTGCCAAG	ATCGGCTGATCCGCAAAGAA
Pka-R1	GCCCTCATCTATGGCACTCC	TCGATTCCCCACAGCTTCAC
Pka-R2	TCAAGGCCTTTGAGCGTTTG	GCGACTCGTAGTCGTCAATGT
LKB1	ATACGCCACGACACTTGGTT	ACCGTGGAGTTGCGGTATTT
CaMKII	CTCGCAGTGCTCTTCAGCTA	TTTGCAGTTTTTACCAAGAACATTT
Atg1(ULK1)	GCCAGCTCCATCGAAAATAACC	GCGGCGCAGCAGGCACAG
Atg5	GCCCCTGCGACTTCACTATCC	CCATTAAATCGGCCAAACTCTTCT
Atg8a	CCAATACAAGGAGGAGCAC	AGGAAGTAGAACTGACCGAC
Atg8b	AATGTGATCCCACCGACATC	TTGAGCGAGTAGTGCCAATG
c-Jun	CAACATGACCGAGGGCTTCT	CCTGCGCTTCCATGTCAATG
ATF-2	CGGAGACATGGATCACCTGG	GATAGCAGGATACGTCGCCC
SMAD4	AACGACATCGGCACCATACA	ACCGTGAGGCACAGCATATT
NFAT	CAAGATCGCGGAACAACACG	CCGAGGACGCCGATATTAGG
Stat	GCGGCTTACTACGAGGAGAA	TCCTGTCTTCGATCCAGGGT
Myc	TGTCCTCGATGTGCTCAACC	GTCACTATCAGAGCCGGTCG
P53	GGAATTGACCACGGAACCCA	ATCCAAAGAGACTTGGGCGG
MEK	ATACGCAAGGCGGAATTGGA	TTGGGCGACGTATTACGCTT
ErK	ATGGTTGTGTCTGCGGATGA	GTTTGGTGTTCAAAGGGCGA
HIF-1α	TCGCTATTTGCCGACGATGG	TGAGGCCAACCAACATGTCA
Pi3K	CCTAATCTGCCTGTTGCCCA	ACTGAGTCGCTTCGTTTCGT
FKBP12	CACCAAGTTCGATTCGTCGC	CGGCTACCGTAGGCATAGTC
PRAS40	CCGGGAGAAATCGGATCAGG	AGCATGGCACGATCGAGTAG
Mitf(TFEB)	GACGGAATCTGGAATCGATTTGG	AGATTTCCCGTTGACGCAGA
